# SOCIAL SUPPORT, HEALTH BEHAVIORS, SELF-ESTEEM, AND SUCCESSFUL AGING IN A SUB-SAHARAN AFRICAN SAMPLE OF OLDER ADULTS

**DOI:** 10.1093/geroni/igae098.1713

**Published:** 2024-12-31

**Authors:** JohnBosco Chukwuorji, Chima Igbokwe, Blessing Ome, Runcie Chidebe, Beatrice Igbokwe, Mary Nwoke, Chidiebere Obioha, Benard Okechi

**Affiliations:** Michigan State University Flint, Flint, Michigan, United States; University of Nigeria Nsukka, Nsukka, Enugu, Nigeria; University of Nigeria Nsukka, Nsukka, Enugu, Nigeria; Miami University, Oxford, Ohio, United States; University of Nigeria Nsukka, Nsukka, Enugu, Nigeria; University of Nigeria Nsukka, Nsukka, Enugu, Nigeria; University of Nigeria Nsukka, Nsukka, Enugu, Nigeria; University of Nigeria Nsukka, Nsukka, Enugu, Nigeria

## Abstract

Previous research demonstrates that social support facilitates successful aging across all cultures. However, the factors that potentially mediate the link between social support and successful aging remain unclear. This study examined whether a healthy lifestyle and self-esteem mediate the association between social support and successful aging. It was hypothesized that the relationship between social support and successful aging would be serially mediated by both healthy lifestyle and self-esteem. Participants were 479 Nigerian retirees (53.4% female; Mean age = 64.81, SD = 6.86). They provided information on relevant demographic variables and completed the following measures: Fantastic Lifestyle Checklist (Fitness Appraisal), Rosenberg Self-esteem Scale, Multi-dimensional Scale of Perceived Social Support Scale, and Successful Ageing Inventory. Three separate regression models (family; friends; and significant other dimensions of social support) were conducted using the Hayes PROCESS macro for SPSS with 5,000 bootstrap estimates. Controlling for age and sex, family support, significant other support, friends support, healthy lifestyle and self-esteem were directly associated with successful aging. The association between family support and successful aging was mediated by healthy lifestyle; and this was also seen for friends’ support and significant other support. The sequential path from social support to successful aging through healthy lifestyle, and then via improved self-esteem, was significant for family support and significant other support, but not friends support. Middle-aged to older adults who have strong support from their families and significant others may be more likely to engage in healthy behaviors and, in turn, experience higher levels of self-esteem, thereby aging well.

